# Study protocol of the GRoningen early-PD Ambroxol treatment (GREAT) trial: a randomized, double-blind, placebo-controlled, single center trial with ambroxol in Parkinson patients with a GBA mutation

**DOI:** 10.1186/s12883-024-03629-9

**Published:** 2024-05-01

**Authors:** O. Siemeling, S. Slingerland, S. van der Zee, T. van Laar

**Affiliations:** 1 Department of Neurology, University Medical Center Groningen, Groningen, The Netherlands; 2 Parkinson Expertise Center Groningen, Groningen, The Netherlands

**Keywords:** Ambroxol, Disease modifying therapy, GBA1, Glucocerebrosidase, Interventional study, Parkinson’s disease

## Abstract

**Background:**

To date, no disease modifying therapies are available for Parkinson’s disease (PD). Since PD is the second most prevalent neurodegenerative disorder, there is a high demand for such therapies. Both environmental and genetic risk factors play an important role in the etiology and progression of PD. The most common genetic risk factor for PD is a mutation in the *GBA1*(GBA)-gene, encoding the lysosomal enzyme glucocerebrosidase (GCase). The mucolytic ambroxol is a repurposed drug, which has shown the property to upregulate GCase activity in-vitro and in-vivo. Ambroxol therefore has the potency to become a disease modifying therapy in PD, which was the reason to design this randomized controlled trial with ambroxol in PD patients.

**Methods:**

This trial is a single-center, double-blind, randomized, placebo-controlled study, including 80 PD patients with a GBA mutation, receiving either ambroxol 1800 mg/day or placebo for 48 weeks. The primary outcome measure is the Unified Parkinson’s Disease Rating Scale motor subscore (part III) of the Movement Disorder Society (MDS-UPDRSIII) in the practically defined off-state at 60 weeks (after a 12-week washout period). Secondary outcomes include a 3,4-dihydroxy-6-18F-fluoro-I-phenylalanine ([^18^F]FDOPA) PET-scan of the brain, Magnetic Resonance Imaging (with resting state f-MRI and Diffusion Tensor Imaging), GCase activity, both intra- and extracellularly, sphingolipid profiles in plasma, Montreal Cognitive Assessment (MoCA), quality of life (QoL) measured by the Parkinson’s Disease Questionnaire (PDQ-39) and the Non-Motor Symptom Scale (NMSS) questionnaire.

**Discussion:**

Ambroxol up to 1200 mg/day has shown effects on human cerebrospinal fluid endpoints, which supports at least passage of the blood-brain-barrier. The dose titration in this trial up to 1800 mg/day will reveal if this dose level is safe and also effective in modifying the course of the disease.

**Trial registration:**

NCT05830396. Registration date: March 20, 2023.

**Supplementary Information:**

The online version contains supplementary material available at 10.1186/s12883-024-03629-9.

## Background

### GBA-PD

Parkinson’s disease (PD) is the second most prevalent neurodegenerative disease and is characterized by various motor- and non-motor problems [1, 2]. Currently, no disease-modifying therapies are available [3]. PD is a multifactorial disorder, with both environmental and genetic risk factors playing an important role in its etiology and progression [4]. The most common genetic risk factor for PD is a mutation in the *GBA1-*gene (GBA), encoding the lysosomal enzyme glucocerebrosidase (GCase) [5]. GCase is a lysosomal hydrolase enzyme responsible for the degradation of the sphingolipid (SL) glucosylceramide (GluCer) into ceramide and glucose. The GBA gene is primarily known for its role in Gaucher’s disease (GD), in which GluCer accumulates in visceral organs [6]. GD is caused by a homozygous variant in the GBA gene. In GD over 400 variants of the GBA gene have been reported. The heterozygous GBA variants will not cause GD, but have shown to increase the risk of developing PD, albeit with a relatively low penetrance [7]. GBA variants are classified according to their clinical manifestation in GD (mild, severe), or the risk they pose for developing PD. Some mutations do not cause GD in homozygous form, but do attribute to the risk for PD [8, 9]. These are referred to as risk variants. Additionally, there are variants of which the clinical significance is unknown. A large nation-wide screening in the Netherlands showed that 15% of the included PD patients carried a GBA mutation, compared to 6,4% in the control group [10]. This screening also showed that over 60% of all mutations are classified as risk variants. PD patients carrying GBA mutations (GBA-PD) tend to have a more severe and rapid progression of both motor and non-motor symptoms [11, 12]. This is not only true for the GD-associated mild and severe variants, but also for risk variants, although the influence of risk factors less clear at this moment [12, 13].

GBA mutations are directly related to loss of GCase activity and lysosomal dysfunction [14, 15]. The exact mechanism behind the reduction of GCase activity remains unclear, although several hypotheses have been formulated [16]. The most widely acknowledged theory concerns a reciprocal relationship between GCase activity and alpha-synuclein aggregation, which plays a central role in the development of PD [17–19]. Other studies, however, could not verify this [20, 21]. Further potential underlying mechanisms include endoplasmic reticulum (ER) stress [22, 23] and deregulation of the autophagic-lysosomal pathway [20, 24].

Reductions in GCase activity may also play a role in sporadic PD, as these individuals have low levels of GCase in the brain and cerebrospinal fluid (CSF) as well [25]. Upregulation of brain cytosolic and lysosomal GCase activity may reduce alpha-synuclein (aSyn) levels, mediating a neuroprotective effect in patients with PD, both with and without GBA mutations [25, 26].

Interestingly, mild GCase deficiencies in GBA-PD do not result in GluCer accumulation, so the lowering of GluCer is unlikely to produce a therapeutic benefit**.** Recent data show that plasma levels of GluCer in GBA-PD and idiopathic PD are similar [27]. Several other studies investigating GluCer levels in CSF and plasma of GBA-PD patients have confirmed that these levels are not increased significantly [28–30]. Moreover, GluCer levels are not elevated in GBA-PD brain tissue [31, 32]. So, GCase mutants have an increased risk of developing PD and may progress more quickly, but this does not seem to be the consequence of an increase in GluCer.

Alternative explanations are related to the fact that GCase plays a role in a complex recycling pathway of SLs, involving various other SLs, besides GluCer. Possibly, mutant GCase has a more widespread effect on this pathway, with a central role for cytosolic ceramide [33]. This cascade is pivotal in the adaptation and reparation of (intra)cellular membranes, involved in synapse formation, neurotransmitter release and autophagy, among others [34]. Therefore, insight into behavior of the SLs involved in this pathway might be of key importance to understand the pathophysiology of GBA-related PD.

### Ambroxol

Ambroxol is a mucolytic expectorant and available on the market in more than 50 countries for over 30 years [35]. Ambroxol acts as a chaperone for GCase, allowing effective trafficking, from the ER and Golgi system to the lysosome [36]. Importantly, ambroxol can be administered orally and crosses the blood-brain barrier [37]. Oral ambroxol has also been demonstrated to increase GCase levels in non-human primates, as well in humans [38–40]. These results support the potential for using ambroxol as a disease-modifying treatment for synucleinopathies, like PD. Studies with ambroxol in humans so far showed that ambroxol can be safely given in those higher dosages for a longer period [39, 41, 42]. Asymptomatic mild proteinuria and increased respiratory mucus production were the only major related adverse reactions [41].

### Dose selection

Participants in this study will receive a maximal dose of 1800 mg/day, using 600 mg capsules, which are titrated with 600 mg per week, up to 1800 mg/day after 3 weeks, if tolerated. This maximal dose was based on previous pharmacological data on ambroxol in humans. One study with PD patients used doses of 1260 mg/day without any serious adverse events [39]. Additionally, a meta-analysis showed that doses of ambroxol up to 30 mg/kg/day to treat pneumonia, were also tolerated very well [43]. Ambroxol has also been administered intravenously in doses up to 20 mg/kg/day, which did not show any adverse events (AEs) [44]. A study in children with oral ambroxol 40 mg/kg/day for 10 days also did not show any AEs. So, the safety profile of ambroxol, even in higher doses, seems to be good.

## Methods/design

### Trial design

This study is a randomized, placebo-controlled trial with ambroxol in non-demented PD patients with a proven GBA mutation. This study is reported following the SPIRIT guidelines (see Supplementary file 1) [45].

**Fig. 1:**
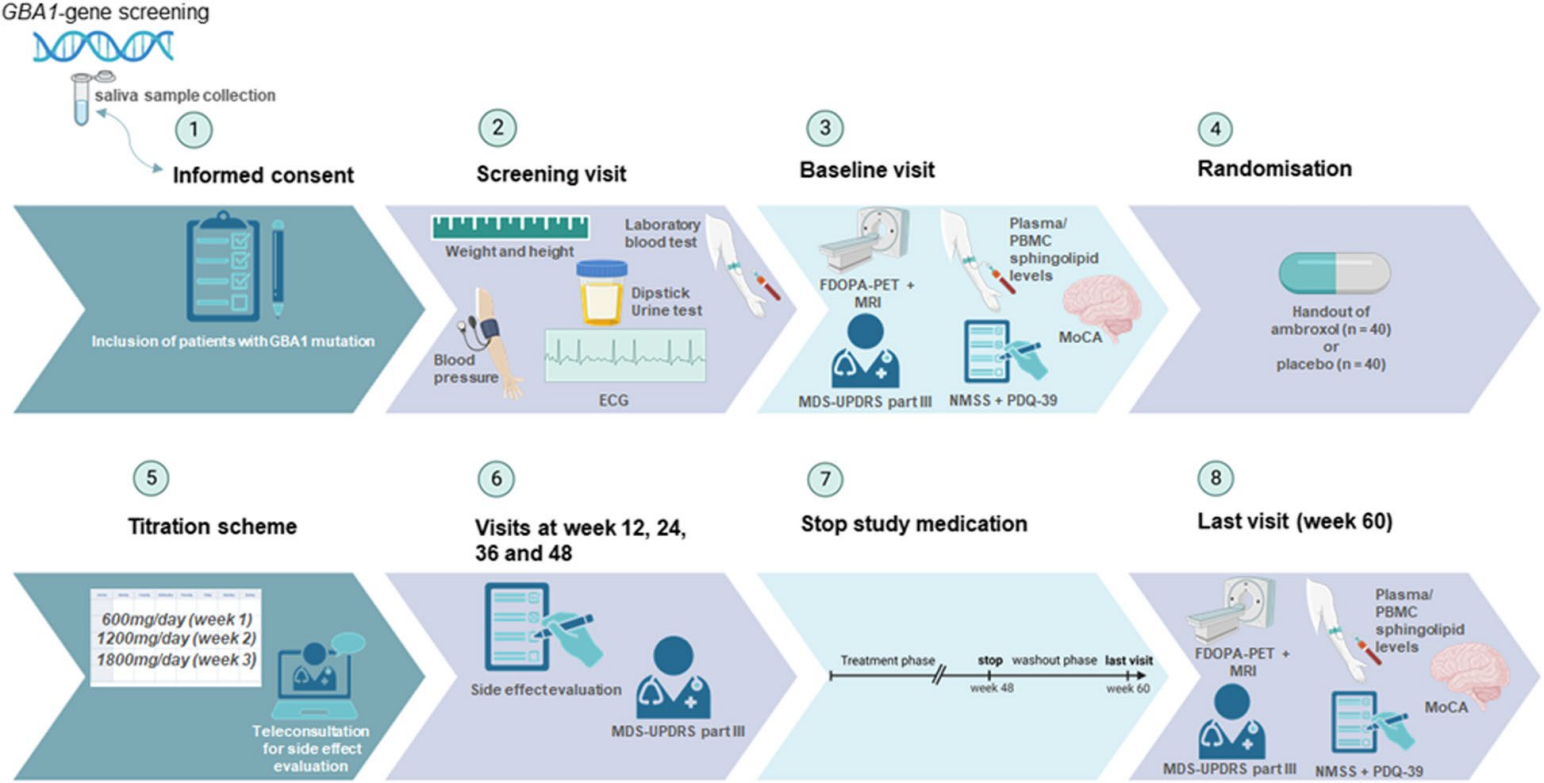
Overview of study visits

This is a single-center trial carried out in the University Medical Center Groningen, with ambroxol versus placebo during 48 weeks, followed by an open 12-week washout period, creating an overall duration of 60 weeks. During these 48 weeks, patients will receive either ambroxol 1800 mg/day or placebo. This includes a 3 week titration period, in which the dose will be increased with 600 mg/day every week. The primary endpoint is the change in off-period scores of the Unified Parkinson’s Disease Rating Scale of the Movement Disorder Society (MDS-UPDRS) part III between baseline and 60 weeks, including a 12-week wash-out period, to exclude an unlikely therapeutic effect of ambroxol. Secondary endpoints are also compared between baseline and 60 weeks, and include safety/tolerability of ambroxol, change in GCase activity in monocytes, striatal F-DOPA uptake as measured by [18]F-DOPA PET scans of the brain, resting state fMRI, related to structural MRI and diffusion tensor imaging (DTI) tractography, quality of life (QoL), measured by the Parkinson’s Disease Questionnaire-39 (PDQ-39), the presence and severity of non-motor symptoms using the Non-Motor Symptoms Scale (NMSS), cognitive changes measured by the MOCA and finally the changes in Levodopa equivalent doses (LED). For an overview of study visits, see Fig. 1.

### Study population

In total, 80 GBA-PD patients will be included in this trial. In order to be included, subjects must have been diagnosed with PD according to Movement Disorders Society (MDS) criteria [46].

Patient’s should have a disease duration of under 10 years at inclusion, and they should be proven carriers of a GBA mutation. Subjects who are unable to undergo an MRI scan or have Deep Brain Stimulation (DBS) are excluded from this study. For a detailed overview of the in- and exclusion criteria, see Table 1.

**Table 1:** In- and exclusion criteria

Inclusion criteria	Exclusion criteria
Diagnosis of PD	Refusal to be informed about an unforeseen clinical finding
Disease duration of < 10 years	Use of DBS
Proven carrier of GBA-mutation	Confirmed dysphagia
Able to write written informed consent	Known sensitivity to study medication
Able to understand protocol	MRI incompatible implants in body
Able to self-administer oral ambroxol	Pregnant or breastfeeding
	Clinically significant medical or surgical condition

#### Recruitment strategy

GBA-PD subjects are selected from a national cohort of GBA-PD patients, identified via a nation-wide screening in the Netherlands, from ongoing GBA screening of the patients of our outpatient PD expertise center and from participants with a GBA mutation of the Dutch Parkinson Cohort study (DUPARC) [47].

Finally, extra GBA-PD participants will be recruited from patients belonging to the Parkinson Platform Northern Netherlands (PPNN), which is a collaborative network of PD treating neurologists in the northern part of the Netherlands. PD patients will be asked to collect a saliva sample for full *GBA* gene sequencing. GBA-carriers will be asked to participate in the study.

### Interventions

The ambroxol capsules of 600 mg were produced exclusively for this study by a Dutch pharma company. Patients will be titrated up to ambroxol 1800 mg/day or placebo (one capsule three times a day) in 3 weeks, starting with 600 mg once daily during the first week, with weekly increases of 600 mg/day. The dosages of the individual subjects will be decreased if serious side effects occur that are probably related to the treatment.

At 48 weeks, all patients stop using either ambroxol or placebo, followed by a 12 week washout period. This washout period ensures the absence of any symptomatic effect of ambroxol.

At 60 weeks, all baseline measurements will be repeated. Participants will continue their regular medication regimen during the study. Dose adaptations of their standard regimens will be allowed, and will be analyzed post-hoc.

### Outcomes

The primary endpoint of this study is the change in the MDS-UPDRS part III motor subscale in the practically defined OFF medication state between baseline and 60 weeks. The MDS-UPDRS part III is a motor scale using a score range from 0 to 132 points, with 0 indicating no disability and 132 indicating total disability. All 18 items are evaluated on a five-point scale from 0 to 4. All items contain clearly formulated instructions for both the patient and the investigator [46, 48]. MDS-UPDRS part III in OFF medication state will be assessed every 12 weeks. OFF medication state is defined as withdrawal from dopaminergic medication for at least 8 hours. To ensure the reliability of the UPDRS III scores, several measures were implemented. First, all examiners had to pass the formal MDS-UPDRS training. All MDS-UPDRS III assessments are video recorded and scored by or together with the main co-investigator.

The study has several secondary endpoints, which are also comparing data at 60 weeks versus baseline scores. The following secondary endpoints will be assessed during this study.

*[* ^*18*^ *F]F-DOPA PET*

The PET tracer [^18^F]F-DOPA PET binds to aromatic amino acid decarboxylase (AADC) and estimates the rate of decarboxylation of [^18^F]F-DOPA to [^18^F]fluorodopamine which corresponds to striatal dopamine production and is considered a reliable tool to assess nigrostriatal dopamine synthesis capacity in vivo [49]. The PET-scan is performed after at least 6 hours of fasting (4 hours for diabetic patients). Participants are premedicated with carbidopa, 60 minutes before receiving 200 MBq of the F-DOPA tracer. The F-DOPA PET scan is performed 90 minutes after injection of the tracer on a Siemens HR+ camera. [^18^F]F-DOPA PET will be performed at baseline and at 60 weeks, to analyze the change in striatal activity. [^18^F]FDOPA striatal-to-occipital ratios of the putamen and caudate nucleus will be calculated to quantify the striatal dopaminergic innervation.

*MRI*

A structural MRI will be performed, which will also serve as the template for PET analysis. In addition, resting state f-MRI will be performed. Fluctuations in the BOLD signal will be used to investigate the functional architecture and connectivity within the brain. Variations in the nigrostriatal projections between the ambroxol and placebo groups will be analyzed using diffusion tensor imaging (DTI) MRI. The microstructural degradation of the nigrostriatal tract in PD patients is associated with the severity of motor symptoms [50]. All MRI sequences will be performed at baseline and 60 weeks.

*GCase activity and SL measurements in mononuclear cells and blood plasma*

The GBA gene encodes the lysosomal enzyme GCase, which catalyzes the hydrolysis of the SL GluCer to produce ceramide. Progression-related single allele mutations are associated with a mild (up to 50%) loss of GCase activity [51]. However, the GluCer levels of PD patients with and without GCase did not show significant differences [27, 30, 31].

So, there are still many questions to be answered about the relationship between lower GCase activity and faster disease progression [52]. Reduced GCase activity may not be related to absolute changes of SLs, but to a reduced flux (the amount of SL recycled per time) through the SL recycling pathway [33]. The recycling SL pathways are required to maintain the availability of cytosolic ceramide, which is needed to adapt and/or repair all kinds of lipid-bilayer membranes in the cell [53]. Unfortunately, measurements of the SL flux are very complex and expensive, and still need validation. Alternatively, static SL levels might give a valuable insight in the effect of ambroxol on the recycling pathway. However, recent data on the biomarker profile of SL in GBA vs non-GBA PD patients did not show any correlation between static intra- and extracellular SL levels. Moreover, only plasma measurements of SL showed differences between GBA-PD, idiopathic PD and healthy volunteers. At the same time, plasma measurements of SL showed the lowest intra-patient variability [54]. Therefore, we choose to perform only static SL measurements in plasma, including all pivotal pathways related to the formation or breakdown of ceramide.

Additionally, the effect of ambroxol on the enzymatic activity of GCase will be measured. GCase activity will be quantified in peripheral blood mononuclear cells (PBMCs), using 5-(Pentafluorobenzoylamino) Fluorescein Di-beta-D-Glucopyranoside (PFB-FDGlu) [55].

The following sphingolipids will be measured (isomers):

- Ceramide (d18:1/18:0)
- Glucosylceramide (d18:1/18:0)
- Galactosylceramide (d18:1/18:0)
- Sphingomyelin (d18:1/18:0)
- Dihydroceramide (d18:1/18:0)
- Ceramide-1-phosphate (d18:1/18:0)
- Sphingosine (d18:1)
- Sphingosine-1-phosphate (d18:1)

**Fig. 2:**
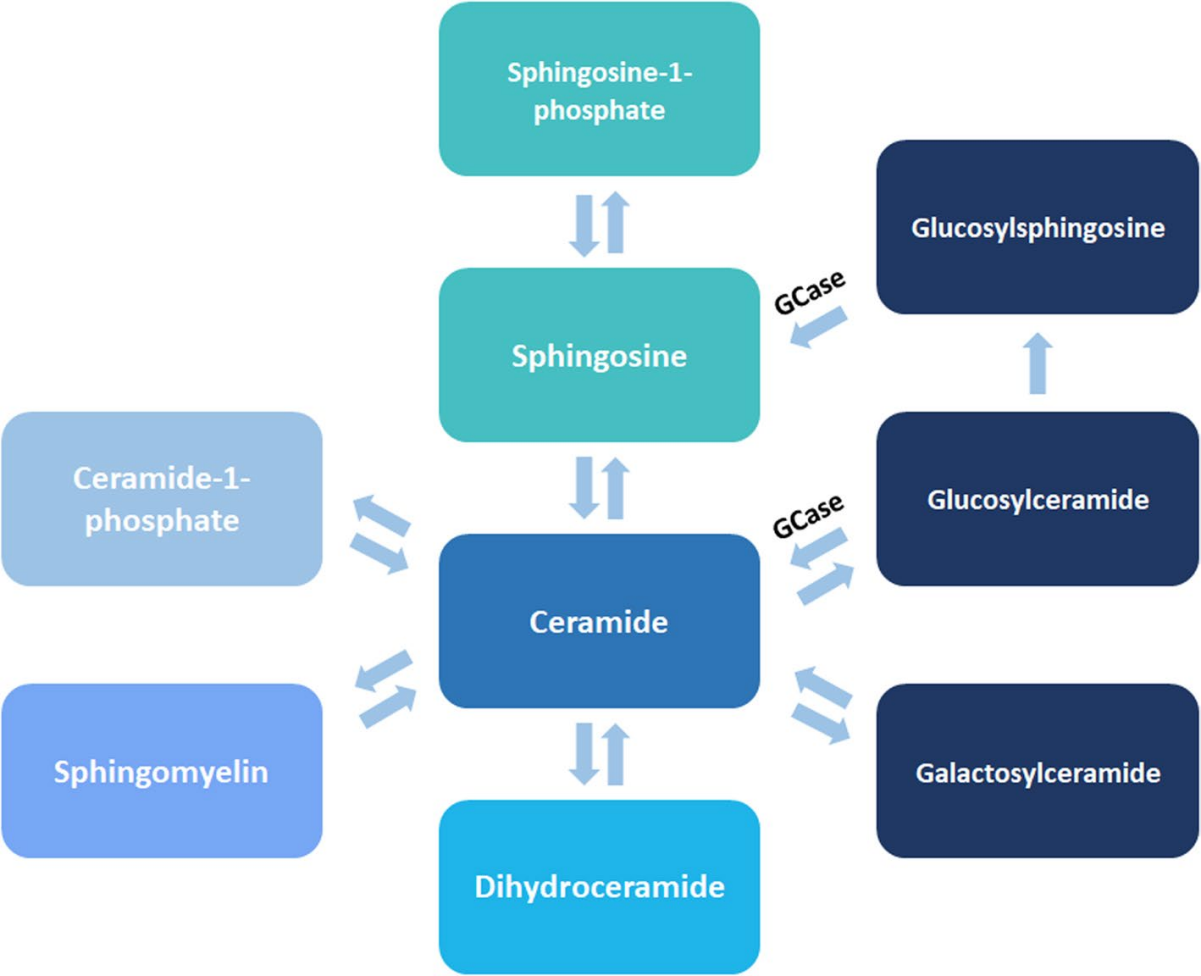
Schematic overview of SL metabolism

Figure 2 shows a schematic overview of part of the SL pathway and the specific measurements performed. Measurements will take place at baseline and after 12, 48 and 60 weeks.

*Montreal cognitive assessment (MoCA)*

Cognitive impairment is a common non-motor symptom in PD and is an important determinant of functional outcome and quality of life. It affects approximately one in four non-demented PD patients [56, 57]. After a 10-year survival, at least 75% of PD patients will develop Parkinson’s disease dementia (PDD) [58]. The cognitive profile of PD is heterogeneous, but impaired executive functioning is the most prominent symptom [59]. The MoCA is widely used as a global assessment of cognitive function. Therefore, it will be assessed in the ON state at baseline and at 60 weeks.

*The non-motor symptoms scale (NMSS)*

The NMSS is a validated 30-item scale to quantitate various non-motor symptoms of PD. All items are rated both in severity (0–3 points, indicating “none”, “mild”, “moderate” and “severe”, respectively) and frequency (1–4 points, indicating “rarely”, “often”, “frequent” and “very frequent”, respectively) with the eventual score per item being the product of those numbers. This adds up to a final score of 0–360 points, with 0 points indicating no experience of symptoms and 360 points indicating maximum experience of symptoms. The NMSS can be used in all stages of disease progression [60]. The NMSS will be assessed in the ON state at baseline and at 60 weeks.

*Parkinson’s disease questionnaire (PDQ-39)*

The PDQ-39 is a 39-item questionnaire to assess the quality of life of PD patients and consists of 8 domains: mobility, activities of daily of living, emotional well-being, stigma, social support, cognition, communication, and bodily discomfort [61]. All items are rated based on a five-point scale (never, occasionally, sometimes, often, always), scoring 0–4 points, respectively. The total score is calculated by adding up all points scored per item. PDQ-39 will be assessed in the ON state at baseline and at 60 weeks.

*Safety*

Side-effects and other AEs will be monitored closely during the trial. For the first 3 weeks, during the titration phase, participants will receive a phone call once a week to assess possible side-effects. They will be asked to report openly first and will then be read out a selection of specific known side-effects of ambroxol to assure thorough reporting. All AEs will be followed until they have abated, or until a stable situation has been reached. Depending on the event, follow up may require additional tests or medical procedures as indicated, and/or referral to the general physician or a medical specialist.

*Other study parameters*

This study will register also the following items to characterize the study population and to monitor side effects.

- Demographics, including age at onset of PD, sex
- LED
- Intoxications: alcohol and drug use
- Intake: six-monthly food diary entailing the last week
- Medical history:
- General medical history
- Neurological comorbidity
- Medical family history

#### Sample size and power calculation

The primary endpoint of this study is the difference in the MDS-UPDRS part III score between the practically defined OFF-state at baseline and 60 weeks, of the ambroxol treated group versus placebo. A previous study with 17 moderate PD patients, treated with high dosages of ambroxol, found an improvement of 6.8 (SD 7.1) points on the MDS-UPDRSIII after 186 days of treatment. However, this was in an open-label non-controlled setting [39].

Ambroxol has never been used for this indication in a double-blind placebo-controlled setting, so insufficient information is available to make a sample size calculation. The effect size was therefore based on collected data about natural disease progression of GBA-PD subjects. Enriching a clinical trial with subgroups of patients, predicted to progress faster on the primary outcome, will considerably reduce the trial size [62]. Several studies calculated the motor progression of GBA-PD. Firstly, GBA-PD patients compared to non-GBA-PD showed a 69% higher decline per year in GBA mutants [62]. Another study reported similar results, with a mean change in the MDS-UPDRS III score per year of 3.4 points (SD 7.7) in GBA carriers. Extrapolating these data to 60 weeks, we postulate an expected difference in MDS-UPDRS-III score of 3.91 points between baseline and 60 weeks in the placebo group. If we would assume no change in MDS-UPDRS III between baseline and 60 weeks in the ambroxol group, a sample size of 80 patients would be required, taking into account a two-sided 5% significance level and a common SD of 6.1, with a power of at least 80% to detect this difference in change of the MDS-UPDRS III score between patients receiving ambroxol and placebo.

### Procedures

#### Screening visit

At screening each patient will undergo a physical examination including measurements of height, weight, pulse and blood pressure. Additional investigations will include electrocardiography, urine dipstick and laboratory blood tests. Women of childbearing potential must have a negative pregnancy test at the screening visit and should use accepted highly effective contraceptive methods.

#### Allocation/randomization

After inclusion, baseline measurements will take place at the University Medical Center Groningen (UMCG). The study drugs (ambroxol and placebo) will be identical in appearance. The capsules will be supplied in brown glass bottles and will only be identifiable by the randomization number. Subjects will be randomized using block randomization and are stratified by sex and disease duration at time of inclusion (0–5 years and 5–10 years). Disease duration will be defined in years and months. ALEA Clinical software will be used for random assignment to placebo or ambroxol [63].

#### Unblinding

Breaking the blind will occur for any participant experiencing a serious adverse event (SAE) for which the clinical management of the SAE will be facilitated by the unblinding of the patient’s treatment allocation. The Principal Investigator (PI) will determine if the patient should be unblinded. All SAEs that are related to the trial medication and are suspected to be unexpected, are submitted to the regulatory agencies within pre-specified timelines. If deemed necessary by the investigator, an unscheduled visit can be performed to discuss study drug discontinuation and the importance of subsequent follow up. As participation in the trial is entirely voluntary, the patient may choose to discontinue trial treatment at any time without any consequences.

### Data collection and monitoring

Patient data are stored in an electronic Case Report Form (eCRF). Data collected during visits will also be stored as a hard copy and will be kept in a file cabinet. Other data, unfit for storage in de eCRF, will be collected in protected servers of the UMCG. All data and documents that are collected during the study will be processed in coded format, with the exception of the Informed Consent Form, on which name and date of birth of the patients are written down.

For a 6-monthly review of study processes, a Data and Safety Monitoring Board (DSMB) has been appointed.

### Statistical analyses

All statistical tests will use an alpha of 0.05, with two-way testing. Data will be analyzed according to the intention-to-treat principle, which will include all patients having completed at least 1 post-randomization follow-up visit. A summary of baseline characteristics and the outcomes at baseline at subsequent treatment-phase visits for the whole cohort will be presented as point estimates with their standard deviations, or as counts and percentages where the data are categorical. Baseline characteristics will be summarized per randomization group.

Primary outcome analyses will evaluate the impact of treatment allocation (ambroxol or placebo) on the difference between MDS UPDRS part III OFF medication scores at 60 weeks follow up. A regression analysis of covariance (ANCOVA) approach will be used to analyze the effect of ambroxol. This is done to adjust for stratification factors and baseline MDS-UPDRS part III values. Also, post-hoc analysis will determine eventual effects of changes in LED on the primary outcome.

Secondary continuous outcomes will be analyzed using regression analyses as well, also adjusting for stratification factors and baseline scores. Comparison of adverse events between treatment groups will be done with χ^2^ tests.

Post-hoc analysis will be performed to correlate GCase activity at baseline and at 60 weeks with MDS-UPDRS III scores. Additionally, the effect of ambroxol on pathological (i.e. severe and mild) GBA variants specifically will be analyzed post-hoc.

#### Missing data

Missing data will be imputed with the last observation-carried forward principle.

### Ethics and dissemination

The GRoningen Early-PD Ambroxol Treatment (GREAT) trial is performed in accordance with the Declaration of Helsinki. The study has been approved by the Medical Research Ethics Committee of the University Medical Center Groningen (METc UMCG) and is registered under METc 2022/179. The study has been reviewed and approved by the Dutch Central Committee on Research involving Human Subjects (CCMO) as competent authority (BI) and is registered under NL77347.042.22. Additionally, the trial is registered at clinicaltrials.gov and is registered under NCT05830396.

Patients received both written and verbal information on the trial before signing the written informed consent.

## Discussion

This study aims to investigate the disease-modifying properties of high-dose ambroxol in PD patients with a GBA mutation. MDS-UPDRS part III in dopaminergic OFF-state is used as the primary outcome measure to investigate the possible disease-modifying effect of ambroxol. Although not perfect, this still is one of the best clinical tools to monitor disease progression [64, 65], in this trial accompanied by imaging markers as well.

Measuring GCase activity in vivo is also a matter of ongoing debate, including the lack of understanding on the precise role of the sphingolipid metabolism and its relationship to PD progression. Our current approach has implemented the most recent findings on sphingolipid metabolism in PD-GBA mutants, using PBMCs (monocytes) and serum sampling [33, 54]. Our sampling strategy represents the major metabolic routes of SLs.

Also, safety and tolerability monitoring are important outcome measures of this study. Side-effects are closely monitored, with extra visits during the titration phase. This allows the investigators to take action on short notice. However, based on previous research, ambroxol has not shown significant safety issues [39, 41, 42].

All PD patients that carry a GBA mutation are allowed in this study. This means that severe, mild and risk variants are included, as well as variants of unknown significance. Based on previous research, it is to be expected that the majority of subjects will be carrier of a risk variant [10]. A post-hoc analysis will be carried out to analyze the effect of ambroxol focused on pathogenic mutations specifically.

Our study uses a washout design, including a 12-week washout period, to eliminate any symptomatic effect of ambroxol.

This trial makes use of capsules, containing 600 mg of ambroxol, which will improve the tolerability and compliance of this treatment.

This is a single-center study, which will improve the homogeneity of the study population. The nation-wide screening in the Netherlands, now complemented with an additional population-wide screening, has resulted in large numbers of PD patients with a known GBA status. There will be an overrepresentation of participants from the northern part of the Netherlands, because the additional screening will take place especially in the neurology practices in the northern counties of the Netherlands.

The intention of this trial is to demonstrate the possible disease-modifying properties of ambroxol. We will have to wait for the trial data, however, in order to see whether these expectations were too optimistic.

### Supplementary Information

**Supplementary Material 1.**

## Data Availability

No datasets were generated or analysed during the current study.

## Abbreviations

AADC: Aromatic amino acid decarboxylase
AE: Adverse event
aSyn: Alpha-synuclein
BI: Competent authority; in Dutch: Bevoegde Instantie
CCMO: Dutch central committee on research involving human subjects; in Dutch: centrale commissie mensgebonden onderzoek
CSF: Cerebrospinal fluid
DBS: Deep brain stimulator
DSMB: Data safety monitoring board
DTI: Diffusion tensor imaging
DUPARC: Dutch Parkinson Cohort study
ECG: Electrocardiogram
eCRF: electronic case report form
ER: Endoplasmic reticulum
[^18^F]F-DOPA: 6-[18F]-fluoro-L-3,4-dihydroxyphenylalanine
GBA-PD: Parkinson patients carrying a *GBA1* mutation
GCase: Glucocerebrosidase
GD: Gaucher disease
GluCer: Glucosylceramide
GREAT: GRoningen early-PD ambroxol treatment
LED: Levodopa equivalent dose
MDS: Movement Disorder Society
METC: Medical research ethics committee (MREC); in Dutch: medisch-ethische toetsingscommissie
MIC: Mild cognitive impairment
MOCA: Montreal cognitive assessment
MRI: Magnetic resonance imaging
NMSS: Non-motor symptoms scale
PBMCs: Peripheral mononuclear blood cells
PD: Parkinson’s disease
PDD: Parkinson’s disease dementia
PDQ-39: 39 item Parkinson’s disease questionnaire
PET: Positron emission tomography
PFD-FDGlu: 5-(Pentafluorobenzoylamino) Fluorescein di-beta-D-glucopyranoside
PI: Principal investigator
PPNN: Parkinson platform Noord Nederland
QoL: Quality of life
(S)AE: (Serious) Adverse event
SL: Sphingolipid
Sponsor: The sponsor is the party that commissions the organisation or performance of the research, for example a pharmaceutical, company, academic hospital, scientific organisation or investigator. A party that provides funding for a study but does not commission it is not regarded as the sponsor, but referred to as a subsidising party
UMCG: University Medical Center Groningen; in Dutch: Universitair Medisch Centrum Groningen
UPDRS: MDS unified parkinson’s disease rating scale
